# Mindfulness to enhance quality of life and support advance care planning: a pilot randomized controlled trial for adults with advanced cancer and their family caregivers

**DOI:** 10.1186/s12904-024-01564-7

**Published:** 2024-09-28

**Authors:** Catherine E. Mosher, Kathleen A. Beck-Coon, Wei Wu, Ashley B. Lewson, Patrick V. Stutz, Linda F. Brown, Qing Tang, Paul R. Helft, Kristin Levoy, Susan E. Hickman, Shelley A. Johns

**Affiliations:** 1 Department of Psychology, Indiana University Indianapolis, 402 North Blackford Street, LD 124, Indianapolis, IN 46202 USA; 2 Indiana University School of Medicine, 1101 West 10th Street, Indianapolis, IN USA; 3 Charles Warren Fairbanks Center for Medical Ethics, Indiana University Health, 1800 North Capitol Avenue, Indianapolis, IN USA; 4 Indiana University Indianapolis Research in Palliative and End of Life Communication and Training Center, 720 Eskenazi Avenue, F2-600, Indianapolis, IN USA; 5 Indiana University Melvin and Bren Simon Comprehensive Cancer Center, Indiana Cancer Pavilion, 535 Barnhill Drive, Suite 473, Indianapolis, IN USA; 6 Department of Community and Health Systems, Indiana University School of Nursing, 600 Barnhill Drive, Indianapolis, IN USA; 7 Indiana University Center for Aging Research, Regenstrief Institute, Inc., 1101 West 10th Street, Indianapolis, IN USA; 8 Center for Health Services Research, Regenstrief Institute, Inc., 1101 West 10th Street, Indianapolis, IN USA

**Keywords:** Neoplasms, Quality of life, Advance care planning, Mindfulness, Family caregivers, Clinical trial

## Abstract

**Background:**

Patients with advanced cancer and family caregivers often use avoidant coping strategies, such as delaying advance care planning discussions, which contribute to deterioration in their quality of life. Mindfulness-based interventions have shown promise in improving quality of life in this population but have rarely been applied to advance care planning. This pilot trial examined the preliminary efficacy of a group-based Mindfulness to Enhance Quality of Life and Support Advance Care Planning (MEANING) intervention for patient-caregiver dyads coping with advanced cancer. Primary outcomes were patient and caregiver quality of life or well-being, and secondary outcomes included patient advanced care planning engagement (self-efficacy and readiness) and other psychological and symptom outcomes.

**Methods:**

In this pilot trial, dyads coping with advanced cancer were recruited from five oncology clinics in the midwestern U.S. and randomized to six weekly group sessions of a mindfulness intervention (*n* = 33 dyads) or usual care (*n* = 22 dyads). Outcomes were assessed via surveys at baseline, post-intervention, and 1 month post-intervention. All available data were included in the multilevel models assessing intervention efficacy.

**Results:**

Patients in the MEANING condition experienced significant increases in existential well-being and self-efficacy for advance care planning across follow-ups, whereas usual care patients did not. Other group differences in outcomes were not statistically significant. These outcomes included other facets of patient well-being, caregiver quality of life, patient readiness for advance care planning, caregiver burden, and patient and caregiver depressive symptoms, anxiety, sleep disturbance, cognitive avoidance, and peaceful acceptance of cancer. However, only MEANING patients showed moderate increases in psychological well-being across follow-ups, and MEANING caregivers showed moderate increases in quality of life at 1-month follow-up. Certain psychological outcomes, such as caregiver burden at 1-month follow-up, also showed moderate improvement in the MEANING condition. Patients in both conditions reported small to moderate increases in readiness to engage in advance care planning.

**Conclusions:**

A mindfulness-based intervention showed promise in improving quality-of-life and advance care planning outcomes in patients and caregivers coping with advanced cancer and warrants further testing.

**Trial Registration:**

ClinicalTrials.gov NCT03257007. Registered 22 August 2017, https://clinicaltrials.gov/ct2/show/NCT03257007.

**Supplementary Information:**

The online version contains supplementary material available at 10.1186/s12904-024-01564-7.

## Background

Many patients with advanced cancer and family caregivers experience increased distress and decrements in quality of life [1–3]. Among caregivers of adults with advanced cancer, greater caregiving burden, or the negative impact of caregiving on various aspects of life, has been associated with reduced quality of life [4]. Distressed adults with advanced cancer and caregivers may employ avoidant coping strategies and have difficulty accepting the illness, which may in turn lead to further distress [5–8].

One avoidant coping strategy is not engaging in advance care planning. While definitions of advance care planning vary, international consensus panels have defined it as the process of supporting adults in exploring values, goals, and preferences to prepare them for future medical decision-making [9, 10]. For patients with serious illnesses like cancer in the United States, advance care planning includes a process of discussions and documenting care preferences on an advance directive or Physician Orders for Scope of Treatment (POST) form [10, 11]. Advance care planning has been associated with earlier and increased use of hospice care [12–15], reduced intensive treatment and hospitalizations at the end of life [13, 15–18], and better quality of life in patients with cancer and caregivers [12, 19]. Despite these benefits, the majority of adults with advanced cancer in the United States do not engage in early advance care planning discussions with their healthcare providers or document care preferences [12, 20]. A variety of factors inhibit advance care planning [21–23] including that patients and caregivers often struggle to accept medical realities [24, 25] and avoid discussions of disease progression or death [23, 26]. Patient and caregiver aversion to the emotional distress surrounding these discussions is addressable [23, 24], but most advance care planning interventions for patients with serious illnesses like cancer fail to address emotional barriers [27, 28]. Rather, they have primarily focused on advance care planning education and traditional communication skills training [27–29], typically producing limited increases in advance care planning discussions and documentation [27].

Mindfulness, or compassionate acceptance of present-moment experiences, is thought to reduce emotional barriers to advance care planning [30]. By increasing distress tolerance or acceptance of unpleasant thoughts and feelings, mindfulness practices may reduce emotional reactivity during end-of-life discussions [31]. Our own single-arm pilot with cancer patient-caregiver dyads was the first to test the impact of a mindfulness-based intervention on advance care planning in any population [32]. Results supported the feasibility, acceptability, and preliminary efficacy of this group-based intervention [32]. Specifically, 59% of eligible patients and 100% of eligible caregivers enrolled and retention rates were high at 1-month follow-up (85% for patients and 92% for caregivers). From baseline to 1-month follow-up, patient engagement in advance care planning nearly doubled, and both patients and caregivers showed large, significant improvement in quality of life. Similarly, other pilot findings suggest that mindfulness-based interventions may improve psychological and quality-of-life outcomes in patients with advanced cancer and caregivers [33–35].

The current randomized pilot trial tests a group-based Mindfulness to Enhance Quality of Life and Support Advance Care Planning (MEANING) intervention that is highly similar to our pilot tested intervention [32]. We examined the impact of MEANING on the quality of life of adults with advanced cancer and their family caregivers relative to usual care. Secondary outcomes included patient advance care planning engagement (self-efficacy, readiness), caregiver burden, and patient and caregiver depressive symptoms, anxiety, sleep disturbance, cognitive avoidance, and peaceful acceptance of cancer. We hypothesized that the MEANING group would show improved outcomes compared to usual care controls.

## Methods

### Study design

Study procedures were approved by the Indiana University institutional review board (IRB#: 1702223546, approved 14 March 2017). Patient-caregiver dyads were randomized to six weekly 2-hour in-person MEANING group sessions or usual care. Outcomes were assessed at baseline, immediately post-intervention, and 1 month post-intervention from April to December 2017.

### Study population

Patient eligibility criteria were as follows: (1) diagnosed with a locally advanced or metastatic solid malignancy at least 3 weeks before enrollment; (2) life expectancy *≤* 12 months according to the attending oncologist [36, 37]; (3) score ≥ 7 on the Mini-Mental Adjustment to Cancer cognitive avoidance subscale [38]; and (4) a consenting family caregiver. Patients were excluded if they (1) scored > 2 on the self-reported Eastern Cooperative Oncology Group measure (indicating they were bedridden or spending most of the day in bed or chair) [39]; (2) showed severe cognitive impairment (*≥* 3 errors on a cognitive screener) [40]; (3) had already completed a POST advance care planning form; or (4) were receiving hospice care. Both patients and caregivers had to be *≥* 18 years of age, fluent in English, and willing and able to travel to the class location for weekly sessions. Although not a study requirement, patients were encouraged to select the caregiver who would serve as their healthcare representative if they became unable to make their own medical decisions.

### Sample

We aimed to recruit 55 dyads and calculated power for 47 dyads at post-intervention (assuming 15% attrition). For each primary outcome, we had 80% power (alpha = 0.05, two-tailed) to detect a large intervention effect (*d* = 0.77) in a linear mixed model [41].

### Recruitment and randomization

Four cohorts of participants were recruited from two medical centers in Indianapolis, Indiana and oncology clinics in three surrounding cities over 16 weeks between March and September 2017. This approach ensured representation from both urban and rural areas. Initial patient eligibility was determined via chart review and consultation with the patient’s oncologist. Research assistants approached potentially eligible patients and caregivers during scheduled clinic visits or by phone at the Indianapolis study sites, whereas patients and caregivers at the other study sites were approached via mailings and phone calls. Interested patients identified their family caregiver and were screened for eligibility. With the patient’s permission, the caregiver was then screened for eligibility. Interested and eligible patients and caregivers attended an enrollment session during which the principal investigator or a research assistant asked them to provide written informed consent and complete the baseline surveys. Most participants completed the baseline survey at the study site. Then dyads (10 to 18 per cohort) were randomized to either the MEANING intervention or usual care and oriented to their assigned group. The enrollment and MEANING sessions were conducted in hospital or research center conference rooms.

The statistician generated a stratified block randomization scheme in Statistical Analysis System (SAS) [42] to balance the groups by the four locations of intervention delivery. Randomly varying block sizes of 2, 4, and 6 were used, and the allocation sequence was concealed from participants and research assistants in opaque sequentially numbered envelopes.

### Measures

Assessments were completed online via Research Electronic Data Capture (REDCap), a secure web platform, or paper surveys at baseline, post-intervention, and 1 month later. At all time points, most participants completed assessments in person at the study sites. Participants also had the option of completing each survey online or on paper at home. Postage-paid envelopes were provided for convenient return of paper surveys. Each person received a $25 gift card per assessment. All outcome measures have shown evidence of reliability and validity. Cronbach’s alphas ranged from 0.71 to 0.95 in this study.

*Primary outcomes*. Patient quality of life was measured with the 16-item McGill Quality of Life Questionnaire (MQoL) [43–46]. In this study, patients completed four MQoL subscales: physical well-being, psychological well-being, existential well-being, and support. Caregiver quality of life was measured with the 35-item Caregiver Quality of Life Index-Cancer (CQoLC) [47]. Items are summed to compute a global quality-of-life score.

*Secondary outcomes*. Patient self-efficacy and readiness for advance care planning were assessed with the 15-item Advance Care Planning Engagement Survey [48, 49]. Self-efficacy items evaluate the patient’s confidence in their ability to ask someone to be a healthcare representative and discuss preferred end-of-life care and flexibility in decision-making with their healthcare representative and doctor. Advance care planning actions (e.g., discussing preferred end-of-life care with their doctor) are elicited within the readiness items, which include the response “I have already done it.” Caregiver burden was assessed with the 12-item Zarit Burden Interview [50]. Patients and caregivers also completed symptom and coping measures, including the 8-item Patient Health Questionnaire depression scale (PHQ-8) [51], the 7-item Generalized Anxiety Disorder scale (GAD-7) [52], the 4-item Patient-Reported Outcomes Measurement Information System (PROMIS) Sleep Disturbance-Short Form [53], the 4-item cognitive avoidance subscale of the Mini-Mental Adjustment to Cancer Scale (mini-MAC) [38], and the 5-item Peaceful Acceptance subscale of the Peace, Equanimity, and Acceptance in the Cancer Experience (PEACE) measure [54].

*Demographic and medical variables*. At baseline, patients and caregivers reported their demographics and completed a checklist of 13 medical conditions adapted from a previous checklist [55]. Patient cancer information was collected by medical record review.

### Study conditions

*MEANING*. While continuing their standard oncology care, MEANING participants attended six weekly 2-hour in-person group sessions led by one of two doctoral-level, certified mindfulness teachers with extensive training from the Center for Mindfulness at the University of Massachusetts. Session components are summarized in Table 1. Both patients and caregivers participated in all session activities and home practice. The course curriculum was adapted from Mindfulness-Based Stress Reduction [56, 57] and Interpersonal Mindfulness programs [58] and featured formal mindfulness meditation training (e.g., body scan, gentle hatha yoga, sitting meditation, compassion meditation). Mindfulness practices facilitate adaptive and non-reactive relating to current thoughts, feelings, and bodily sensations. Mindfulness practice adaptations were offered to those with severe illness. For example, if awareness of breath proved difficult for participants with dyspnea, attention was focused on other sensations (e.g., noticing sounds). For participants unable to stand for yoga, chair adaptations and supine stretching options were offered and modeled by the teacher. Yoga mats and cushions were available for all participants. Participants were given audio recordings of each mindfulness practice covered in class (15-minute body scan, 15-minute sitting meditation, 20-minute yoga). Shorter lovingkindness and compassion practices were taught in class. Participants were encouraged to practice at home 15–20 min per day, 6 days per week.

**Table 1:** Description of MEANING intervention sessions

	Session Theme	Mindfulness Practices	Didactics	Home Practice
**1**	Awareness: Meeting ourselves where we are in honesty and kindness	• Mindful eating (raisin exercise) • Body scan	• Introduction and guidelines • Define mindfulness • Introduce mindful speaking and listening skills	• Body scan daily • Eat one meal mindfully • Complete one daily activity mindfully
**2**	Perception and creative responding: Wholeness no matter what is here	• Body scan • Hatha yoga stretching • Awareness of breath • Sitting meditation	• Role of perception, conditioning, and other factors in stress appraisal • Meeting struggle with compassion and non-judgment • Mindfulness to face challenging aspects of life	• Alternate body scan and yoga daily • Sitting meditation: 10 min daily • Optional body scan before sleep • Daily calendar of pleasant events
**3**	Relational presence: Mindful communication skills and hospitality toward the self	• Sitting meditation • Hatha yoga stretching • Mindful communication	• Physiological and psychological bases of stress reactivity • Mindful communication: pause, relax, open, allow • Compassion as attitude and behavior • Relating to self and others	• Choice of daily mindfulness practice • Daily calendars of reactivity-responsivity and communication
**4**	Mindful communication: Cultivating compassion in speech and action; advance care planning as empowerment	• Sitting meditation • Hatha yoga stretching • Mindful communication • Lovingkindness practice	• Mindful communication about: (1) change and uncertainty, (2) goals of care with providers and family caregivers • Education about advance care planning, including POST form and palliative care programs	• Choice of daily mindfulness practice • Read ASCO advance care planning booklet • Review POST form together in mindful dialogue
**5**	Mindful communication amid challenging thoughts and feelings	• Sitting meditation • Hatha yoga stretching • Mindful communication • Lovingkindness practice	• Using mindful communication guidelines, engage in deeper discussion of advance care planning • Discuss benefits of timely advance care planning • Review advance care planning tools (e.g., POST form)	• Choice of daily mindfulness practice • Mindful communication in everyday life • Reflect on skills learned
**6**	The rest of your life: Making the practice your own	• Body scan • Hatha yoga stretching • Sitting meditation • Lovingkindness practice	• Discuss growth in adapting to cancer-related challenges • Using mindful communication skills, invite each person to share what has been learned • Invite patients to discuss care preferences with oncology team and sign the POST form if ready • Review core mindfulness skills	• Mindfulness resources handout

MEANING = Mindfulness to Enhance Quality of Life and Support Advance Care Planning; ASCO = American Society of Clinical Oncology; POST = Physician Orders for Scope of Treatment

In sessions 4–6, the teacher guided dyads in practicing mindful speaking and listening skills [59] and provided advance care planning education. Participants received the *American Society of Clinical Oncology’s Advanced Cancer Care Planning: A Decision-Making Guide for Patients and Families Facing Serious Illness* booklet. Specific advance care planning tools, including the Indiana POST form, were also provided with guidance on appropriate use. Class discussion honored diversity in beliefs and values, including informed refusal of advance care planning.

The mindfulness teachers were supervised on a weekly basis by a board-certified clinical health psychologist or a certified mindfulness teacher. Three doctoral-level certified mindfulness teachers reviewed a randomly selected 50% of sessions for adherence to the manual using checklists (Additional file 1). Across mindfulness cohorts, the mean fidelity rating was 100% (number of required topics and practices covered in each session/total number of criteria). Raters also evaluated each mindfulness teacher’s capacity to embody and facilitate the qualities and practice of mindfulness [60–62] (15 items per session), and the mean mindfulness facilitation skill rating was 98.1%. The psychologist provided feedback on treatment fidelity and quality.

*Usual care*. Participants assigned to usual care continued to receive their standard care from their oncology team. Contact information for the oncology social worker at their cancer center was provided. After completing the 1-month follow-up, usual care participants met with a mindfulness teacher for one hour and received the same guided audio recordings of mindfulness as the MEANING group, information about mindfulness meditation and trainings available in the community, and advance care planning resources available at their cancer center and online.

### Statistical analyses

Using *t*-tests and Fisher’s exact tests, baseline comparisons of study conditions were conducted for patients and caregivers separately. An intent-to-treat framework was employed for data analyses. Multilevel models (MLMs) were used to assess the preliminary efficacy of the MEANING intervention, accounting for repeated measures. For outcomes applying only to patients or caregivers, the MLMs included main and interaction effects of study condition and time (baseline, post-intervention, and 1 month post-intervention; treated as categorical). For example, this MLM approach was used to evaluate intervention effects on quality of life due to differences in its assessment between patients and caregivers. Indeed, small to moderate correlations were found between patient and caregiver quality-of-life measures at baseline (*r*s = 0.24–0.38), post-intervention (*r*s = 0.17–0.36), and 1 month post-intervention (*r*s = 0.14–0.37).

For outcomes that were identical for patients and caregivers, MLMs for dyadic data were used [63, 64]. In dyadic models, fixed-effects parameters included all main effects and two- and three-way interaction effects among study condition, time, and role (patient vs. caregivers). Intervention effects are evidenced by a significant condition-by-time interaction. The three-way interaction among study condition, time, and role indicated the degree to which intervention effects differed for patients and caregivers. Random-effects parameters included separate residual variances for patients and caregivers and the covariance between the residuals which indicates the similarity in the two partners’ scores at a certain time point after taking into account the fixed effects. Random intercepts for dyads were also included to model variance in the mean outcome across dyads. Two-tailed *p-*values < 0.05 were considered statistically significant. A partial correlation coefficient (*pr*), computed based on the *F* value and degrees of freedom, was the effect size measure for each fixed effect [65].

As a supplemental analysis, Cohen’s *d*s were computed for within-group and between-group effects on outcomes for participants who completed surveys at all three time points. The *d* for a within-group effect was calculated as the average difference between baseline and each follow-up divided by the standard deviation (SD) of the change. The *d* for a between-group effect was calculated as the difference between average changes for each condition divided by the pooled SD of the change.

## Results

### Participant characteristics

**Fig. 1:**
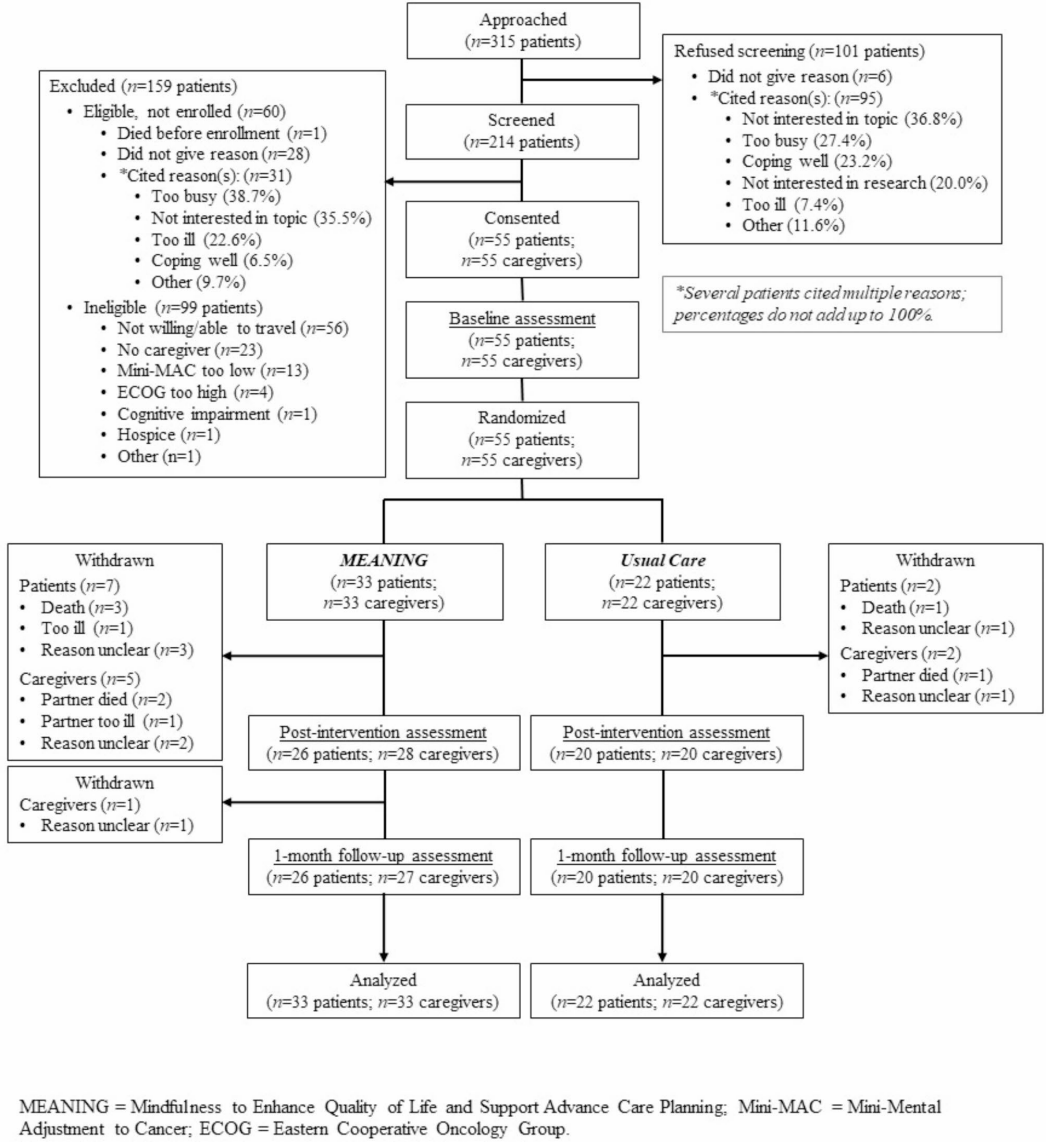
Consolidated Standards of Reporting Trials (CONSORT) Diagram

Of the 315 patients who were approached, 214 (68%) agreed to be screened for eligibility (see Fig. 1). Of those screened, 99 were found to be ineligible, 1 died before enrollment, and 59 were eligible but declined to participate. All 55 approached caregivers agreed to participate and, thus, 55 patient-caregiver dyads were enrolled and randomized to either the MEANING intervention (*n* = 33 dyads) or usual care (*n* = 22 dyads). MEANING participants attended a mean of 4.2 of the 6 sessions, with 70% of dyads attending at least 5 of the 6 sessions. Retention was strong with 83.6% of patients and 85.5% of caregivers completing the 1-month follow-up. Retention did not significantly vary by study condition.

Participant characteristics and comparisons by study condition at baseline are presented in Table 2. Demographics, medical factors, and outcomes did not differ by study condition at baseline, except for patient depressive symptoms and patient and caregiver cognitive avoidance. At baseline, MEANING patients had greater depressive symptoms than control patients, and MEANING patients and caregivers had lower cognitive avoidance than controls.

**Table 2:** Patient and caregiver characteristics and group comparisons at baseline

Characteristics	Patients (*n* = 55)		t-test/Fisher’s Exact Test *p*	Caregivers (*n* = 55)		t-test/Fisher’s Exact Test *p*
	MEANING (*n* = 33)	Usual Care (*n* = 22)		MEANING (*n* = 33)	Usual Care (*n* = 22)	
Gender, *n* (%)			0.82			0.40
Male	13 (39)	8 (36)		15 (45)	7 (32)	
Female	20 (61)	14 (64)		18 (55)	15 (68)	
Age			0.82			0.44
Mean	70.6	71.25		66.7	65.8	
SD	9.0	12.2		14.3	13.2	
Range	47.6–85.3	43.8–88.2		33.0-87.9	37.8–90.8	
Race, *n* (%)			1.00			1.00
White	31 (94)	21 (95)		31 (94)	20 (91)	
Black	2 (6)	1 (5)		1 (3)	1 (5)	
Other	0 (0)	0 (0)		1 (3)	0 (0)	
Ethnicity, *n* (%)			0.56			1.00
Non-Hispanic/Latinx	32 (97)	20 (91)		32 (97)	21 (95)	
Hispanic/Latinx	1 (3)	2 (9)		1 (3)	1 (5)	
Employment status, *n* (%)			0.59			0.46
Employed full or part-time	10 (30)	7 (32)		11 (33)	10 (45)	
Retired	15 (45)	11 (50)		16 (48)	7 (32)	
Unable to work	5 (15)	4 (18)		1 (3)	3 (14)	
Other	3 (9)	0 (0)		2 (6)	0 (0)	
Household income US$, *n* (%)			0.08			0.34
≤$49,999	12 (36)	12 (55)		14 (42)	8 (36)	
$50,000 - $100,000	11 (33)	5 (23)		10 (30)	6 (27)	
>$100,000	9 (27)	5 (23)		8 (24)	7 (32)	
Education, *n* (%)			0.66			0.22
No bachelor’s degree	14 (42)	11 (50)		15 (45)	11 (50)	
Bachelor’s degree	6 (18)	5 (23)		5 (15)	3 (14)	
Graduate degree	13 (39)	6 (27)		13 (39)	7 (32)	
Caregiver relationship to the patient, *n* (%)						0.88
Spouse/partner	---	---		22 (67)	13 (59)	
Other family member or friend	---	---		11 (33)	9 (41)	
Married, *n* (%)	25 (76)	16 (73)	0.64	28 (85)	18 (82)	0.99
Cancer type, *n* (%)			0.26			
Breast	8 (24)	8 (36)		---	---	
Prostate	4 (12)	2 (9)		---	---	
Colorectal	2 (6)	5 (23)		---	---	
Melanoma	3 (9)	2 (9)		---	---	
Lung	2 (6)	2 (9)		---	---	
Pancreatic	3 (9)	1 (5)		---	---	
Other (e.g., esophageal, head/neck, ovarian, renal)	11 (33)	2 (9)		---	---	
Cancer stage, *n* (%)			0.22			
III	6 (18)	1 (5)		---	---	
IV	27 (82)	21 (95)		---	---	
Treatments received, *n* (%)			0.64			
Chemotherapy	17 (52)	12 (55)		---	---	
Hormonal therapy	6 (18)	2 (9)		---	---	
Immunotherapy	7 (21)	4 (18)		---	---	
Radiation	3 (9)	0 (0)		---	---	
Number of comorbidities			0.97			0.35
Mean	1.88	1.86		1.09	1.45	
SD	1.56	1.49		0.98	1.63	
Range	0–6	0–6		0–3	0–5	

MEANING = Mindfulness to Enhance Quality of Life and Support Advance Care Planning

For certain characteristics, sample sizes do not add up to 33 (MEANING) or 22 (usual care) due to missing data or no ongoing treatment.

### Primary outcomes

Results of MLM analyses showed no condition-by-time interaction effects on patient or caregiver quality-of-life outcomes, except for patient existential well-being (*p* = 0.03, *pr* = 0.20; Table 3). The pattern of means in Table 3 shows improved existential well-being in MEANING patients at both follow-ups, whereas the mean scores for control patients remain relatively stable.

**Table 3:** Results from multilevel linear models (*N* = 55 dyads)

Outcome Fixed Effect	MEANING Intervention			Usual Care			df	*F*	*p*	*Pr*	*95% CI Pr*	
	Baseline	Post-intervention	1 Month Post-intervention	Baseline	Post-intervention	1 Month Post-intervention						
	Mean (SD)	Mean (SD)	Mean (SD)	Mean (SD)	Mean (SD)	Mean (SD)						
Primary Outcomes:												
PT QoL: physical well-being	5.64 (2.66)	6.76 (1.64)	6.15 (2.41)	6.77 (2.00)	7.00 (1.95)	7.35 (2.03)						
Group							45.42	3.55	0.07	0.27	0.00	0.54
Time							86.51	1.46	0.24	0.13	-0.08	0.34
Group x time							86.51	0.79	0.46	0.10	-0.11	0.30
PT QoL: psychological well-being	6.40 (2.73)	7.26 (2.20)	7.60 (1.98)	7.67 (2.01)	7.76 (1.70)	8.13 (1.81)						
Group							46.32	2.96	0.09	0.25	-0.03	0.52
Time							84.31	3.88	0.02	0.21	0.01	0.41
Group x time							84.31	0.53	0.59	0.08	-0.13	0.29
PT QoL: existential well-being	6.26 (1.82)	7.27 (1.40)	7.17 (1.64)	7.39 (1.22)	7.23 (1.29)	7.71 (1.28)						
Group							50.37	3.29	0.08	0.25	-0.01	0.51
Time							87.23	5.19	0.01	0.24	0.04	0.44
Group x time							87.23	3.78	**0.03**	0.20	0.00	0.40
PT QoL: support	7.56 (1.66)	7.88 (1.58)	7.94 (1.57)	8.07 (1.37)	7.78 (1.78)	8.23 (1.20)						
Group							49.50	0.48	0.49	0.10	-0.18	0.37
Time							88.19	0.79	0.46	0.09	-0.11	0.30
Group x time							88.19	1.02	0.37	0.11	-0.10	0.31
CG global QoL	2.48 (0.52)	2.60 (0.44)	2.76 (0.42)	2.63 (0.52)	2.70 (0.42)	2.70 (0.51)						
Group							51.32	0.99	0.32	0.14	-0.13	0.41
Time							90.97	4.43	0.01	0.22	0.02	0.41
Group x time							90.97	1.10	0.34	0.11	-0.09	0.31
**Secondary Outcomes:**												
PT ACP self-efficacy	3.33 (0.57)	3.49 (0.70)	3.60 (0.58)	3.65 (0.50)	3.60 (0.56)	3.40 (0.83)						
Group							53.20	0.50	0.48	0.10	-0.17	0.36
Time							92.93	0.21	0.81	0.05	-0.16	0.25
Group x time							92.93	3.53	**0.03**	0.19	0.00	0.39
PT ACP readiness	3.25 (1.29)	3.48 (1.05)	3.62 (1.16)	3.16 (1.45)	3.64 (1.22)	3.60 (1.45)						
Group							52.65	0.02	0.88	0.02	-0.25	0.29
Time							90.20	6.53	0.00	0.26	0.07	0.45
Group x time							90.20	0.41	0.66	0.07	-0.14	0.27
CG burden	10.33 (6.64)	10.04 (5.15)	8.57 (6.68)	7.77 (5.35)	8.95 (6.45)	8.55 (5.64)						
Group							51.24	1.35	0.25	0.16	-0.11	0.43
Time							91.00	0.89	0.41	0.10	-0.11	0.30
Group x time							91.00	0.76	0.47	0.09	-0.11	0.29
PT depressive symptoms	7.45 (5.12)	5.52 (4.97)	6.88 (5.90)	4.32 (4.02)	4.10 (4.14)	3.26 (3.31)						
CG depressive symptoms	4.91 (4.89)	3.58 (4.07)	3.78 (3.51)	3.62 (4.20)	5.90 (5.44)	4.65 (5.58)						
Group							49.13	2.40	0.13	0.22	-0.05	0.48
Time							93.80	0.16	0.85	0.04	-0.16	0.24
Role							47.95	0.80	0.38	0.13	-0.15	0.41
Time x role							92.47	2.20	0.12	0.15	-0.05	0.35
Group x time							93.80	2.97	0.06	0.18	-0.02	0.37
Group x role							47.95	3.36	0.07	0.26	-0.01	0.52
Group x time x role							92.47	0.64	0.53	0.08	-0.12	0.29
PT anxiety	4.06 (4.30)	3.00 (3.45)	3.77 (4.80)	2.57 (2.87)	2.53 (3.52)	2.37 (2.69)						
CG anxiety	4.64 (3.77)	3.76 (3.35)	3.85 (3.77)	3.52 (5.17)	4.95 (5.56)	4.40 (6.19)						
Group							50.34	0.42	0.52	0.09	-0.18	0.36
Time							87.54	0.28	0.76	0.06	-0.15	0.27
Role							53.13	3.46	0.07	0.25	-0.01	0.50
Time x role							88.72	0.30	0.74	0.06	-0.15	0.27
Group x time							87.54	1.52	0.22	0.13	-0.08	0.34
Group x role							53.13	0.58	0.45	0.10	-0.16	0.37
Group x time x role							88.72	0.05	0.96	0.02	-0.19	0.23
PT sleep disturbance	8.88 (2.22)	8.84 (2.56)	8.73 (2.34)	8.32 (1.64)	8.25 (1.80)	8.70 (2.47)						
CG sleep disturbance	11.47 (2.26)	11.38 (1.92)	11.04 (1.87)	12.05 (2.27)	12.42 (2.59)	11.70 (2.49)						
Group							51.54	0.23	0.64	0.07	-0.21	0.34
Time							89.77	0.30	0.74	0.06	-0.15	0.26
Role							49.17	75.61	0.00	0.78	0.67	0.89
Time x role							85.65	1.38	0.26	0.13	-0.08	0.33
Group x time							89.77	0.31	0.73	0.06	-0.15	0.26
Group x role							49.17	2.07	0.16	0.20	-0.07	0.47
Group x time x role							85.65	0.61	0.55	0.08	-0.13	0.29
PT cognitive avoidance	8.76 (2.74)	8.64 (2.40)	8.46 (3.17)	10.52 (2.79)	10.10 (3.35)	9.95 (2.87)						
CG cognitive avoidance	7.36 (3.21)	7.22 (2.58)	7.44 (2.33)	8.45 (2.77)	8.90 (2.75)	9.30 (2.92)						
Group							50.68	6.58	0.01	0.34	0.10	0.58
Time							91.71	0.04	0.96	0.02	-0.18	0.22
Role							54.28	10.08	0.00	0.40	0.17	0.62
Time x role							96.56	1.71	0.19	0.13	-0.06	0.33
Group x time							91.71	0.04	0.97	0.02	-0.18	0.22
Group x role							54.28	0.03	0.87	0.02	-0.24	0.29
Group x time x role							96.56	0.43	0.65	0.07	-0.13	0.27
PT peaceful acceptance	3.28 (0.55)	3.51 (0.46)	3.43 (0.61)	3.45 (0.46)	3.46 (0.45)	3.44 (0.51)						
CG peaceful acceptance	3.05 (0.63)	3.24 (0.54)	3.14 (0.62)	3.06 (0.62)	3.07 (0.71)	3.03 (0.63)						
Group							54.24	0.00	0.96	0.01	-0.26	0.27
Time							90.80	2.16	0.12	0.15	-0.05	0.35
Role							52.10	19.22	0.00	0.52	0.32	0.72
Time x role							93.05	0.18	0.83	0.04	-0.16	0.25
Group x time							90.80	1.60	0.21	0.13	-0.07	0.33
Group x role							52.10	1.13	0.29	0.15	-0.12	0.41
Group x time x role							93.05	0.00	1.00	0.00	-0.20	0.21

ACP = advance care planning; CG = caregiver; MEANING = Mindfulness to Enhance Quality of Life and Support Advance Care Planning; *Pr =* partial correlation; PT = patient; QoL = quality of life. Significant *p*-values for group x time interactions are in bold.

### Secondary outcomes

MLM analyses showed a significant condition-by-time interaction effect for patient advance care planning self-efficacy (*p* = 0.03, *pr* = 0.19). Mean levels of advance care planning self-efficacy showed small improvements in MEANING patients and small decreases in control patients (Table 3). There were no condition-by-time interaction effects on patient readiness to engage in advance care planning or caregiver burden. Additionally, results from the dyadic analyses showed no two-way or three-way interaction effects among condition, time, and role for depressive symptoms, anxiety, sleep disturbance, cognitive avoidance, or peaceful acceptance of cancer.

### Supplemental analyses of survey completers

Among survey completers, patient psychological well-being showed moderate improvement in the MEANING condition at both follow-ups (*d*s = 0.33, 0.50) and little change in control patients (*d*s = 0.04, 0.17; Additional file 2). MEANING patients also reported large to moderate improvements in existential well-being at both follow-ups (*d*s = 0.86, 0.71), whereas control patients reported little change (*d*s=-0.16, 0.24). Additionally, MEANING patients’ physical well-being showed a moderate increase post-intervention (*d* = 0.42) that was not sustained 1 month later (*d* = 0.16), and patient perceptions of support showed limited change in both study conditions (*d*s=-0.15 to 0.25). For caregivers, quality of life moderately improved in the MEANING condition at 1 month post-intervention (*d* = 0.60), whereas controls reported little change in quality of life at both follow-ups (*d*s = 0.21, 0.20; Additional file 3). Effect sizes for secondary outcomes are found in Additional files 2 and 3. Patient advance care planning self-efficacy and certain psychological outcomes (e.g., patient and caregiver peaceful acceptance, caregiver burden) only showed improvement in the MEANING condition. Patients in both study conditions showed small to moderate increases in advance care planning readiness.

## Discussion

This is the first randomized trial testing a mindfulness-based intervention to support advance care planning. While previous advance care planning interventions have included training in advance care planning options or traditional communication skills [27–29], our MEANING intervention is a blend of advance care planning education and mindfulness skills to address emotional barriers to advance care planning. Patients in the MEANING condition showed increases in advance care planning self-efficacy over time, whereas these improvements were not observed in the usual care group. However, patients in both conditions reported small to moderate increases in readiness to engage in advance care planning across follow-ups. Completing study surveys may have heightened patients’ awareness of the importance of advance care planning. Thus, during the study, patients in both conditions showed increased readiness or contemplation and preparation for advance care planning, but only MEANING patients showed greater self-efficacy for this behavior, a key correlate of advance care planning behaviors [66]. Growth in mindfulness skills, such as maintaining an open and accepting posture towards difficult thoughts and feelings, may have led to increased self-efficacy for engaging in end-of-life discussions among MEANING participants.

Patients in the MEANING condition also showed increased existential well-being over time, whereas usual care controls showed little change in this outcome. Although other differences in outcomes between study conditions were not statistically significant, MEANING patients showed moderate increases in psychological well-being across follow-ups, and MEANING caregivers showed moderate increases in quality of life at 1-month follow-up. Certain psychological outcomes, such as patient anxiety and depressive symptoms post-intervention, caregiver burden at 1-month follow-up, and patient and caregiver peaceful acceptance, showed moderate improvement in the MEANING condition. Usual care participants either reported little change or worsening of these outcomes. Our results converge with prior pilots showing beneficial effects of mindfulness-based interventions on psychological and quality-of-life outcomes in patients with advanced cancer and caregivers [33–35].

Several factors may help explain the positive impact of mindfulness-based interventions on psychological and quality-of-life outcomes. First, mindfulness practices increase distress tolerance by facilitating compassionate awareness of thoughts and feelings [31]. Additionally, maintaining an open, accepting posture toward thoughts and feelings may interrupt maladaptive reactions to these experiences, such as rumination and catastrophizing, which then allows for greater focus on activities that improve quality of life. Finally, engaging in mindful communication skills with their family member, a key component of our intervention, allows for a shared understanding of the illness, resulting in choices that enhance quality of life [30]. For instance, patients may share their preferred course of action, and caregivers may be relieved to know patient preferences regarding their medical care.

Study limitations warrant mention. The sample was primarily white and receiving care at oncology clinics in Indiana. The data were collected in 2017, and responsibilities of the PI resulted in a delay in submitting the findings for publication. However, the topic remains highly relevant in the United States and other countries where advance care planning is underutilized. Additionally, the small sample size limited statistical power for detecting significant small to moderate effects; however, our primary goal was to obtain preliminary estimates of intervention effects prior to conducting a fully powered trial. This trial may include a longer follow-up period and an active control, such as advance care planning education without training in mindfulness skills. Caregivers’ awareness of the patient’s plans for end-of-life care may also be assessed in future research.

## Conclusions

Our preliminary results suggest that training in mindfulness skills and advance care planning may improve quality-of-life, advance care planning, and psychological outcomes in patients and caregivers coping with advanced cancer. Next steps include testing the intervention in a large-scale randomized trial and examining mechanisms, such as increased distress tolerance, underlying the intervention’s effects. Demonstrating the intervention’s efficacy with large, diverse samples will support its widespread dissemination and implementation in cancer care. Additionally, results will lay the groundwork for mindfulness-based intervention trials addressing emotional barriers to advance care planning in other populations with serious illnesses.

## Electronic supplementary material

Below is the link to the electronic supplementary material.

Additional File 1: Checklists for skill ratings and fidelity monitoring (Tables showing the checklists used for mindfulness facilitation skill ratings and fidelity monitoring)

Additional File 2: Patient outcomes (Table with descriptive statistics and effect sizes for outcomes for patients who completed study surveys)

Additional File 3: Caregiver outcomes (Table with descriptive statistics and effect sizes for outcomes for caregivers who completed study surveys)

## Data Availability

The datasets generated during and/or analyzed during the current study are available from Dr. Shelley A. Johns on reasonable request (email: sheljohn@iu.edu). Intervention materials and the full trial protocol may also be requested from Dr. Johns.

## Abbreviations

CQoLC: Caregiver Quality of Life Index-Cancer
GAD-7: 7-item Generalized Anxiety Disorder scale
MEANING: Mindfulness to Enhance Quality of Life and Support Advance Care Planning
mini-MAC: Mini-Mental Adjustment to Cancer Scale
MLMs: Multilevel models
MQoL: McGill Quality of Life Questionnaire
PEACE: Peace, Equanimity, and Acceptance in the Cancer Experience
PHQ-8: 8-item Patient Health Questionnaire
POST: Physician Orders for Scope of Treatment
*pr*: Partial correlation coefficient
PROMIS: Patient-Reported Outcomes Measurement Information System
SAS: Statistical Analysis System
SD: Standard deviation
