# Supplementary material for: Mindfulness to enhance quality of life and support advance care planning: a pilot randomized controlled trial for adults with advanced cancer and their family caregivers
Source: BMC Palliat Care. 2024 Sep 28;23:232. doi: 10.1186/s12904-024-01564-7 (PMC11439323; doi:10.1186/s12904-024-01564-7)
Supplement: Supplementary file 1 — Additional File 1: Checklists for skill ratings and fidelity monitoring (Tables showing the checklists used for mindfulness facilitation skill ratings and fidelity monitoring) [file 12904_2024_1564_MOESM1_ESM.docx]

| **Ratings are on 0-2 scale: 0=No Evidence; 1= Slight/Some Evidence; 2 = Definite Evidence** | | | | | | | |
| --- | --- | --- | --- | --- | --- | --- | --- |
| **Basic Session Components** | **Class 1** | **Class 2** | **Class 3** | **Class 4** | **Class 5** | **Class 6** | **Total**  **(range 0-12 per row)** |
| Facilitator presents mindfulness processes in meditation, mindful movement, inquiry, and/or dialogue on contemplation topics and specific didactic teaching points arising both from the framework of the class theme and from participant experiences. |  |  |  |  |  |  |  |
| Facilitator encourages participants’ involvement in sharing on home practice and in-class experiential exercises as well as dyadic and large group sharing. |  |  |  |  |  |  |  |
| Facilitator leads mindfulness practices at the beginning and end of each session and incorporates a mindful approach throughout the session. |  |  |  |  |  |  |  |
| Facilitator encourages a flexible approach, adapting to and meeting the needs that may arise in any given session. |  |  |  |  |  |  |  |
| Facilitator explains rationale for home practice assignments and gives specific instructions for completing the lessons for the next group session. |  |  |  |  |  |  |  |
| **General Guidelines for Facilitation** | | | | | | | |
| Facilitator encourages participants to engage how mindfulness meets their everyday lives. |  |  |  |  |  |  |  |
| Facilitator guides discussion, encourages curiosity, and tolerates discomfort by inviting all to turn towards whatever is being experienced including participants’ painful feelings, without jumping in to rescue or change what is being experienced. |  |  |  |  |  |  |  |
| Facilitator asks open-ended questions and requests clarification/more explanation to promote discussion among group members using experiential exercises and inquiry based in participants’ experience over lecturing. |  |  |  |  |  |  |  |
| Facilitator maintains a mindful stance with all practice guidance (e.g. including but not exclusive of raisin practice, body scan, yoga, sitting meditation, compassion meditation) coming directly out of the practice of the facilitator who is practicing with the participants. |  |  |  |  |  |  |  |
| Facilitator embodies a warm, equal, vulnerable, compassionate, genuine, and respectful approach. |  |  |  |  |  |  |  |
|  | **Class 1** | **Class 2** | **Class 3** | **Class 4** | **Class 5** | **Class 6** | **Total**  **(range 0-12 per row)** |
| Facilitator embodies a present-moment focus in guiding formal practices and informal inquiry, adapting practices to the physical and emotional needs of those in the room. |  |  |  |  |  |  |  |
| Facilitator self-discloses when in the best interest of the participants in recognizing that she/he shares in our common humanity. |  |  |  |  |  |  |  |
| Facilitator encourages participants’ exploration of their relationship to experience more than content or opinion. |  |  |  |  |  |  |  |
| In meditation guidance, the facilitator uses allowing and inviting rather than directive language with use of options for practice and gerunds that point to present moment experiencing. |  |  |  |  |  |  |  |
| Facilitator asks permission to explore more deeply, particularly focusing on a body-centered awareness. For example, “Are you willing to look at this together to see what we can learn?” “What are you noticing in the body right now?” Inquiry often includes an invitation to all in the room in the exploration and invites in other perspectives. |  |  |  |  |  |  |  |
| **TOTAL** (range 0-30 for each column) |  |  |  |  |  |  | /180 |

| **Specific Class Curriculum Elements**  Rated 0-1: 0= element is NOT present; 1= element IS present | |
| --- | --- |
| **Session 1** | **Rating**  **(0-1)** |
| 1. Welcome and remind purpose of group. |  |
| 1. Remind definition of mindfulness as non-judgmental, kind attention. |  |
| 1. Setting ground rules as a trusting learning community including confidentiality. |  |
| 1. Mindful eating as an introduction to everyday mindfulness practice. |  |
| 1. Learning Body Scan as means to ground one’s experience in how the body is in any moment. |  |
| 1. Explanation of home practice and provision of materials, including home practice logs. |  |
| 1. Closing practice—Awareness of breath. |  |
| **Session 2** | **Rating**  **(0-1)** |
| 1. Body scan practice bringing in working with mind-body challenges and struggles. |  |
| 1. Introduce seeing thoughts as a process rather than getting lost in their content. |  |
| 1. Introduction of mindful stretching as a means of self-care and mindful self-knowledge. |  |
| 1. Beginning to explore the role of perception and judgment in the origins of suffering, focusing on well-being, rather than disease. |  |
| 1. Explanation of home practice and provision of materials. |  |
| 1. Closing practice—Awareness of breath. |  |
| **Session 3** | **Rating**  **(0-1)** |
| 1. Sitting meditation highlighting self-compassion in allowing whatever is here in the present moment. |  |
| 1. Review of pleasant events as experience in the body and mind. |  |
| 1. Didactic on stress reactivity and effects of mindfulness in times of stress. |  |
| 1. Introduce practices of mindfulness and compassion “on the spot” in times of challenge. |  |
| 1. Exploring mindfulness practice in relationship, introducing the first three mindful dialogue guidelines. |  |
| 1. Explanation of home practice and provision of materials. |  |
| 1. Closing mindfulness practice. |  |

| **Session 4** | **Rating**  **(0-1)** |
| --- | --- |
| 1. Exploring triggers of reactivity in communication. |  |
| 1. Expand compassion practice in communication. |  |
| 1. Use dialogic mindfulness practice to share on life changes and resistance to change. 2. Introduce advanced care planning (ACP) educational materials and Physician Orders for Scope of Treatment (POST) form as tools of empowerment, care, and an opportunity for clear communication of each individual’s understanding of what is valued near the end of life. |  |
|  |  |
| 1. Explanation of home practice and provision of materials. |  |
| 1. Closing practice—Lovingkindness. |  |
| **Session 5** | **Rating**  **(0-1)** |
| 1. Reinforce and expand compassion practice. |  |
| 1. Dialogic inquiry on what gets in the way of meaningful conversation on challenging topics and how mindfulness practice supports connection and communication in the face of these challenges. |  |
| 1. Group discussion on experience of sharing on ACP tools and POST pointing to empowerment, care, and connection. |  |
| 1. Explanation of home practice and provision of materials. |  |
| 1. Closing practice—Expanded lovingkindness practice. |  |
| **Session 6** | **Rating**  **(0-1)** |
| 1. Practice reinforcement of all mindfulness practices from class (i.e., body scan, yoga, compassionate sitting practice). |  |
| 1. Dialogic and group exploration of how mindfulness practice may be supportive going forward in empowering choices and connection in relationships. |  |
| 1. Support materials for after the class. |  |
| 1. Closing practice—Lovingkindness. |  |
| **TOTAL across all sessions (range 0-35)** |  |

**SUMMARY of RATINGS**

| Basic Session Components/General Guidelines Across All Sessions (0-180) |  |
| --- | --- |
| Specific Class Curriculum Elements Across All Sessions (0-35) |  |
| **TOTAL (0-215)** |  |
