# Supplementary material for: Mindfulness to enhance quality of life and support advance care planning: a pilot randomized controlled trial for adults with advanced cancer and their family caregivers
Source: BMC Palliat Care. 2024 Sep 28;23:232. doi: 10.1186/s12904-024-01564-7 (PMC11439323; doi:10.1186/s12904-024-01564-7)
Supplement: Supplementary file 2 — Additional File 2: Patient outcomes (Table with descriptive statistics and effect sizes for outcomes for patients who completed study surveys) [file 12904_2024_1564_MOESM2_ESM.docx]

| Additional file 2. Descriptive statistics and effect sizes for patient outcomes. | | | | | | | |  |  |
| --- | --- | --- | --- | --- | --- | --- | --- | --- | --- |
|  | **MEANING Intervention ^b^** | | | **Usual Care ^c^** | | |  |  |  |
| Outcome ^a^ | **Mean change** | **SD change** | **Within- Group ES** | **Mean change** | **SD change** | **Within-**  **Group ES** | **Between-Groups ES,**  **(95% CI)** | | |
| Primary Outcomes: |  |  |  |  |  |  |  |  |  |
| QoL: Physical well-being |  |  |  |  |  |  |  |  |  |
| Baseline to Follow-up 1 | 1.12 | 2.70 | 0.42 | 0.23 | 2.34 | 0.10 | 0.36 | -0.23 | 0.95 |
| Baseline to Follow-up 2 | 0.52 | 3.24 | 0.16 | 0.58 | 2.78 | 0.21 | -0.02 | -0.61 | 0.57 |
|  |  |  |  |  |  |  |  |  |  |
| QoL: Psychological well-being |  |  |  |  |  |  |  |  |  |
| Baseline to Follow-up 1 | 0.86 | 2.61 | 0.33 | 0.09 | 2.09 | 0.04 | 0.33 | -0.26 | 0.92 |
| Baseline to Follow-up 2 | 1.20 | 2.39 | 0.50 | 0.45 | 2.60 | 0.17 | 0.30 | -0.29 | 0.89 |
|  |  |  |  |  |  |  |  |  |  |
| QoL: Existential well-being |  |  |  |  |  |  |  |  |  |
| Baseline to Follow-up 1 | 1.02 | 1.19 | 0.86 | -0.16 | 1.00 | -0.16 | 1.08 | 0.49 | 1.67 |
| Baseline to Follow-up 2 | 0.91 | 1.28 | 0.71 | 0.31 | 1.29 | 0.24 | 0.47 | -0.12 | 1.06 |
|  |  |  |  |  |  |  |  |  |  |
| QoL: Support |  |  |  |  |  |  |  |  |  |
| Baseline to Follow-up 1 | 0.32 | 1.41 | 0.23 | -0.29 | 1.95 | -0.15 | 0.37 | -0.22 | 0.96 |
| Baseline to Follow-up 2 | 0.38 | 1.51 | 0.25 | 0.16 | 1.81 | 0.09 | 0.14 | -0.45 | 0.73 |
|  |  |  |  |  |  |  |  |  |  |
| Secondary Outcomes: |  |  |  |  |  |  |  |  |  |
| Advance care planning self-efficacy |  |  |  |  |  |  |  |  |  |
| Baseline to Follow-up 1 | 0.16 | 0.58 | 0.28 | -0.05 | 0.43 | -0.12 | 0.41 | -0.18 | 1.00 |
| Baseline to Follow-up 2 | 0.27 | 0.36 | 0.74 | -0.25 | 0.91 | -0.28 | 0.80 | 0.21 | 1.39 |
|  |  |  |  |  |  |  |  |  |  |
| Advance care planning readiness |  |  |  |  |  |  |  |  |  |
| Baseline to Follow-up 1 | 0.23 | 0.81 | 0.29 | 0.48 | 1.33 | 0.36 | -0.24 | -0.83 | 0.35 |
| Baseline to Follow-up 2 | 0.37 | 0.86 | 0.43 | 0.44 | 1.21 | 0.36 | -0.07 | -0.66 | 0.52 |
| Additional file 2. Continued. | | | | | | | |  |  |
|  | **MEANING Intervention ^b^** | | | **Usual Care ^c^** | | |  |  |  |
| Outcome ^a^ | **Mean change** | **SD change** | **Within- Group ES** | **Mean change** | **SD change** | **Within-**  **Group ES** | **Between-Groups ES,**  **(95% CI)** | | |
| Depressive symptoms |  |  |  |  |  |  |  |  |  |
| Baseline to Follow-up 1 | -1.93 | 5.64 | -0.34 | -0.22 | 3.65 | -0.06 | -0.36 | -0.95 | 0.23 |
| Baseline to Follow-up 2 | -0.57 | 6.49 | -0.09 | -1.06 | 3.96 | -0.27 | 0.09 | -0.50 | 0.68 |
|  |  |  |  |  |  |  |  |  |  |
| Anxiety |  |  |  |  |  |  |  |  |  |
| Baseline to Follow-up 1 | -1.06 | 3.19 | -0.33 | -0.05 | 2.50 | -0.02 | -0.35 | -0.94 | 0.24 |
| Baseline to Follow-up 2 | -0.29 | 5.22 | -0.06 | -0.20 | 2.97 | -0.07 | -0.02 | -0.61 | 0.57 |
|  |  |  |  |  |  |  |  |  |  |
| Sleep disturbance |  |  |  |  |  |  |  |  |  |
| Baseline to Follow-up 1 | -0.04 | 2.42 | -0.02 | -0.07 | 2.07 | -0.03 | 0.01 | -0.58 | 0.60 |
| Baseline to Follow-up 2 | -0.15 | 2.43 | -0.06 | 0.38 | 2.24 | 0.17 | -0.23 | -0.82 | 0.36 |
|  |  |  |  |  |  |  |  |  |  |
| Cognitive avoidance |  |  |  |  |  |  |  |  |  |
| Baseline to Follow-up 1 | -0.12 | 3.13 | -0.04 | -0.42 | 2.62 | -0.16 | 0.11 | -0.48 | 0.70 |
| Baseline to Follow-up 2 | -0.30 | 3.17 | -0.09 | -0.57 | 2.31 | -0.25 | 0.10 | -0.49 | 0.69 |
|  |  |  |  |  |  |  |  |  |  |
| Peaceful acceptance |  |  |  |  |  |  |  |  |  |
| Baseline to Follow-up 1 | 0.23 | 0.45 | 0.52 | 0.01 | 0.34 | 0.02 | 0.56 | -0.03 | 1.15 |
| Baseline to Follow-up 2 | 0.15 | 0.28 | 0.55 | -0.02 | 0.44 | -0.04 | 0.48 | -0.11 | 1.07 |
| QoL = quality of life; MEANING = Mindfulness to Enhance Quality of Life and Support Advance Care Planning; ES = Effect Size; CI = Confidence Interval.  ^a^ Effect sizes are Cohen’s *ds*. Follow-ups 1 and 2 occurred immediately post-intervention and 1 month post-intervention, respectively.  ^b^ Data from survey completers were analyzed (*n=* 25 at follow-up 1 and *n=* 26 at follow-up 2).  ^c^ Data from survey completers were analyzed (*n=* 20 at follow-up 1 and *n=* 20 at follow-up 2). | | | | | | | | | |
