# Supplementary material for: Mindfulness to enhance quality of life and support advance care planning: a pilot randomized controlled trial for adults with advanced cancer and their family caregivers
Source: BMC Palliat Care. 2024 Sep 28;23:232. doi: 10.1186/s12904-024-01564-7 (PMC11439323; doi:10.1186/s12904-024-01564-7)
Supplement: Supplementary file 3 — Additional File 3: Caregiver outcomes (Table with descriptive statistics and effect sizes for outcomes for caregivers who completed study surveys) [file 12904_2024_1564_MOESM3_ESM.docx]

| Additional file 3. Descriptive statistics and effect sizes for caregiver outcomes. | | | | | | | |  |  |
| --- | --- | --- | --- | --- | --- | --- | --- | --- | --- |
|  | **MEANING Intervention ^b^** | | | **Usual Care ^c^** | | | **Between-Groups ES** | | |
| Outcomes ^a^ | **Mean change** | **SD change** | **Within- Group ES** | **Mean change** | **SD change** | **Within-**  **Group ES** | **ES** | **95% CI** | |
| Primary Outcome: |  |  |  |  |  |  |  |  |  |
| Global quality of life |  |  |  |  |  |  |  |  |  |
| Baseline to Follow-up 1 | 0.12 | 0.42 | 0.29 | 0.07 | 0.32 | 0.21 | 0.14 | -0.44 | 0.72 |
| Baseline to Follow-up 2 | 0.27 | 0.45 | 0.60 | 0.07 | 0.34 | 0.20 | 0.51 | -0.08 | 1.09 |
| Secondary Outcomes: |  |  |  |  |  |  |  |  |  |
| Caregiver burden |  |  |  |  |  |  |  |  |  |
| Baseline to Follow-up 1 | -0.30 | 5.37 | -0.06 | 1.18 | 6.09 | 0.19 | -0.26 | -0.84 | 0.32 |
| Baseline to Follow-up 2 | -1.77 | 4.57 | -0.39 | 0.78 | 3.56 | 0.22 | -0.62 | -1.21 | -0.02 |
| Depressive symptoms |  |  |  |  |  |  |  |  |  |
| Baseline to Follow-up 1 | -1.33 | 5.95 | -0.22 | 2.28 | 2.99 | 0.76 | -0.75 | -1.35 | -0.14 |
| Baseline to Follow-up 2 | -1.13 | 5.09 | -0.22 | 1.03 | 3.38 | 0.31 | -0.49 | -1.08 | 0.1 |
| Anxiety |  |  |  |  |  |  |  |  |  |
| Baseline to Follow-up 1 | -0.88 | 4.09 | -0.21 | 1.43 | 4.17 | 0.34 | -0.56 | -1.15 | 0.04 |
| Baseline to Follow-up 2 | -0.78 | 4.38 | -0.18 | 0.88 | 5.00 | 0.18 | -0.36 | -0.94 | 0.23 |
| Sleep disturbance |  |  |  |  |  |  |  |  |  |
| Baseline to Follow-up 1 | -0.08 | 1.92 | -0.04 | 0.37 | 2.29 | 0.16 | -0.22 | -0.8 | 0.37 |
| Baseline to Follow-up 2 | -0.43 | 1.90 | -0.23 | -0.35 | 2.52 | -0.14 | -0.04 | -0.62 | 0.54 |
| Cognitive avoidance |  |  |  |  |  |  |  |  |  |
| Baseline to Follow-up 1 | -0.14 | 2.28 | -0.06 | 0.45 | 2.35 | 0.19 | -0.26 | -0.84 | 0.32 |
| Baseline to Follow-up 2 | 0.08 | 2.09 | 0.04 | 0.85 | 2.68 | 0.32 | -0.33 | -0.91 | 0.25 |
| Peaceful acceptance |  |  |  |  |  |  |  |  |  |
| Baseline to Follow-up 1 | 0.19 | 0.41 | 0.46 | 0.01 | 0.53 | 0.01 | 0.40 | -0.18 | 0.98 |
| Baseline to Follow-up 2 | 0.09 | 0.60 | 0.15 | -0.04 | 0.36 | -0.11 | 0.25 | -0.33 | 0.83 |
| MEANING = Mindfulness to Enhance Quality of Life and Support Advance Care Planning; ES = Effect Size; CI = Confidence Interval. | | | | | | | | | |
| ^a^ Effect sizes are Cohen’s *ds*. Follow-ups 1 and 2 occurred immediately post-intervention and 1 month post-intervention, respectively.  ^b^ Data from survey completers were analyzed (*n* = 33 at follow-up 1 and *n* = 27 at follow-up 2).  ^c^ Data from survey completers were analyzed (*n =*22 at follow-up 1 and *n =*20 at follow-up 2). | | | | | | | | | |
